# Current Advances in Meat Nutritional, Sensory and Physical Quality Improvement

**DOI:** 10.3390/foods9030321

**Published:** 2020-03-10

**Authors:** Mohammed Gagaoua, Brigitte Picard

**Affiliations:** 1 Université Clermont Auvergne, INRAE, VetAgro Sup, UMR Herbivores, 63122 Saint-Genès-Champanelle, France; 2 Food Quality and Sensory Science Department, Teagasc Ashtown Food Research Centre, Ashtown, Dublin 15, Ireland

**Keywords:** meat science, carcass and meat qualities, sensory and technological quality, muscle biochemistry, statistical tools for meat quality prediction, OMICs tools, production systems

## Abstract

Meat is an important source of proteins, vitamins, minerals and fat, and these nutrients are important for their beneficial effects on human health. In recent years, meat quality has become a more relevant topic for consumers with regard to health and sensory characteristics, and for beef industry stakeholders because it affects their profitability. Therefore, the control of meat quality, including technological, sensory and nutritional quality traits, constitutes an important target for any farm animal production. What those qualities are and how we best evaluate them at the different levels of the continuum from the farm to fork are critical to understanding meat production and consumption patterns. However, despite the efforts of the industrials to control the eating and nutritional quality traits of meat and meat products, there remains a high level of variability, which is one reason for consumer dissatisfaction. This Special Issue focuses on the study of continuum aspects from farm to fork, which would have an impact on the control of the nutritional, sensory and technological aspects of carcass, muscle, meat and meat-product qualities. It groups fourteen original studies and one comprehensive review within five main topics that are (i) production systems and rearing practices, (ii) prediction of meat qualities, (iii) statistical approaches for meat quality prediction/management, (iv) muscle biochemistry and proteomics techniques and (v) consumer acceptability, development and characterisation of meat products.

Meat quality constitutes the main topic for both consumers with regard to health and sensory characteristics and beef industry stakeholders for economic considerations. Despite the efforts of beef sector actors to control the eating and nutritional quality of beef, there remains a high level of variability in these quality traits, which is one reason for consumer dissatisfaction. However, it is recognised that science and innovation will play a great role in helping the industry respond to consumer concerns and expectations. Accordingly, and within the idea and objective of bringing together original studies dealing with the continuum aspects, i.e., from farm to fork [1,2], having an impact on the nutritional, sensory and technological aspects of carcass, muscle, meat and meat-product qualities, we edit this Special Issue on “current advances in meat nutritional, sensory and physical quality improvement”. From the fifteen published papers, five main research topics were covered: (i) production systems and rearing practices, (ii) prediction of meat qualities, (iii) statistical approaches for meat quality prediction/management, (iv) muscle biochemistry and proteomics techniques and (v) consumer acceptability, development and characterisation of meat products. 

In the first topic dealing with production systems and rearing practices and their relationships with carcass and meat qualities, almost six papers can be categorized [3,4,5,6,7,8]. The first study by Moran et al. [3] compares the quality of beef from bulls slaughtered at 15 months of age and reared in typical Irish indoor production systems with those raised in novel grass-based systems. This study was conducted in response to some European markets seeking bulls to be slaughtered at less than 16 months of age with a carcass fatness score of six on the 15-scale. The context of the study by Moran and co-workers, therefore, was to compare cheaper alternative systems with those demonstrated to achieve the carcass weight and fat cover specifications. The authors found that the only grass-based system that reached the current market requirement was the ration based on grass silage and concentrates, offered indoors with associated costs of housing. Therefore, the production of late-maturing sired bulls for slaughter at less than 16 months of age from pasture does not seem to be an option for meeting current market requirements. It is worthwhile to note that the beef-eating quality of grass-fed animals was not detrimentally affected. The second study is by Couvreur et al. [4], which has been performed on the protected designation of origin (PDO) Maine–Anjou cull cows. The authors investigate the relationships among the characteristics of cull beef cows, rearing practices and physicochemical characteristics and sensory traits of two differing muscles, *Longissimus thoracis* (LT) and *Rectus abdominis* (RA). The novelty of this study is the consideration of the interaction between animal type and finishing practices at the farm scale on the final meat quality based on a clustering approach that distinguishes different rearing practices. The authors found that finishing practices, whatever the cluster, have less effect than animal type on RA and LT meat properties. Also, the effects observed on meat quality are directly related to farmers’ practices and provide new advice and modifications in culled cows rearing practices that would help improve the meat quality of PDO Maine–Anjou. In the same context, two studies by Soulat et al. [5,6] investigate using the same experimental design on 96 heifers of the protected geographical indication (PGI) Fleur d’Aubrac (crossbreed Charolais × Aubrac); first, the effect of the rearing managements applied during the heifers’ whole life on carcass and flank steak quality [5], and second, the effect on five muscles from ribs in the chuck sale section [6]. To achieve their goals, the authors conducted surveys with farmers to identify the main rearing managements applied, allowing them to select the animals with controlled slaughter and post-slaughter conditions to limit any effects on the final meat quality. In the first preliminary study, rearing management applied during the heifers’ whole life seem to have more impact on carcass traits than on the flank steak properties. The authors conclude that carcass traits could be improved without altering the meat properties. In the second study, the authors investigate the impact of four different rearing managements (identified by a statistical clustering method) on the traits of the LT muscle (sensory, rheological, and colour) and the rheological traits of four other muscles, *complexus*, *infraspinatus*, *rhomboideus*, and *serratus ventralis,* in the ribs of the chuck sale section. It seems from the main results that the whole life period of the animals has no effect on tenderness (sensory or rheological analyses) of the rib muscles. The findings of these two studies by Soulat et al. [5,6] together with those of Couvreur et al. [4] demonstrate that it is possible to obtain similar meat qualities with different rearing managements. The fifth study is by Cafferky et al. [7] and it investigates, on a big cattle database at industrial scale of crossbred bull and steer progeny, the effect of breed (eight beef sire breeds representative of the Irish herd: Aberdeen Angus, Belgian Blue, Charolais, Hereford, Limousin, Parthenaise, Salers and Simmental) and gender on meat quality traits (Warner–Bratzler shear force (WBSF), intramuscular fat (IMF%), cook-loss%, drip-loss%, colour (L*, a*, b*) and ultimate pH) of *Longissimus thoracis* et *lumborum* (LTL) muscle. The authors report on animals obtained and reared under the same feeding and environmental conditions that the sire breed had a significant effect on IMF%, cook-loss% and drip-loss%. With respect to breed, Aberdeen Angus-sired progeny had the highest IMF% and the lowest drip-loss%, Limousin-sired offspring had the lowest cook-loss%, while Belgian Blue- and Parthenaise-sired progeny scored the highest for drip-loss%. On another hand, the comparisons of bulls and steers highlight that castration significantly impacts WBSF, IMF% and cook-loss%. Steers in comparison to bulls had higher IMF% and reduced WBSF and cook-loss%, implying steer beef to be more tender and juicy, with more favourable IMF%. This study supports the hypothesis that breed and gender influence the final eating qualities of beef. Finally, the sixth study grouped in this first topic is by Chartrin et al. [8] on turkey meat. It evaluates the meat produced from breeder turkeys in comparison with that of standard turkeys. In line with the objectives of the Special Issue, the technological, nutritional, and sensorial quality of breasts and thighs with drumsticks of turkey male and female breeders was characterized in comparison to that of drumsticks of growing male and female turkeys from the Grademaker line. The authors prove that the differences that exist between males and females, and between standard and breeder turkeys, are mainly a consequence of differences in the age at slaughter and due to sexual dimorphism on body weight. The meat of female breeders had characteristics close to those of standard turkeys, whereas the meat of male breeders was clearly distinguishable, particularly by displaying lower tenderness and water-holding capacity.

In the second topic grouping of studies within the prediction of meat qualities, two papers by Berri et al. [9] and Sahar et al. [10] are published. The former is a review titled “Predicting the Quality of Meat: Myth or Reality?”. The authors provide an overview of recent advances made in the field of meat quality prediction, particularly in Europe. The different approaches applied at the laboratory or at the production scale for the development of equations and tools using biological (genomic or phenotypic) or physical (spectroscopy) sources are extensively discussed. The authors develop the recent strategies and findings in Europe related to (i) the search of genes that control the quality of pork and chicken meat, (ii) the recent advances in the search and quantification of protein biomarkers that predict or explain beef tenderness, (iii) the potential of blood biomarkers to predict meat quality by giving the first encouraging results on the use of high-resolution nuclear magnetic resonance or NMR, followed by (iv) the potential of spectroscopic methods. For this last approach, the authors review that the most significant progress was achieved using near-infrared spectroscopy (NIRS) to predict the composition and nutritional value of meats. However, it is worthwhile to note that predicting the functional properties of meats using these methods, especially the sensory quality, is slightly difficult, and further studies are needed. Finally, the example of the Meat Standards Australia phenotypic model, which predicts the eating quality of beef based on a combination of upstream and downstream data, is briefly described. In frame of the objectives of this second topic and more specifically on the use of spectroscopic methods, the research paper by Sahar et al. [10] illustrate the potential of visible–near-infrared (Vis–NIR) spectroscopy to predict physicochemical quality traits (ultimate pH, colour, cook loss and drip loss) in a big dataset of 368 samples of bovine LTL muscle. The authors highlight that the application of Vis–NIR spectroscopy directly on the meat carcass is advantageous as it does not require the preparation of the sample before analysis, and it is applicable to the prediction of quality online using a fibre-optic probe.

In the third topic that groups papers dealing with statistical approaches for meat quality prediction/management, three original papers are published [11,12,13] and they are complimentary to the previous topic by addressing some of the objectives reported by Berri et al. [9] for the development of prediction/management tools of beef qualities. The first study by Ellies-Oury et al. [11] presenta a new methodology for the selection of protein biomarkers of tenderness in five different bovine muscles using a multi-block model: the data-driven sparse partial least square. In the same context, Gagaoua et al. [12] present an innovative approach for the prediction of beef tenderness by a combination of statistical methods that are “chemometrics” and “supervised learning” to manage the integrated data of the continuum from the farm to fork and select the potential predictors of beef tenderness. Among a total of 60 variables, including WBSF and belonging to 4 levels of the continuum that are farm–slaughterhouse–muscle–meat, partial least squares (PLS) and three decision tree methods (C&RT, classification and regression tree; QUEST, quick, unbiased, efficient regression tree; and CHAID, chi-squared automatic interaction detection) were tested to select the driving factors of WBSF and propose predictive decision tools using the selected variables. In the last study, Conanec et al. [13] propose a new method to manage the trade-off between four performance goals that are the nutritional and organoleptic properties of meat and cattle performances, including carcass properties. Among their findings, the authors show that there is no antagonism between organoleptic quality and nutritional quality. Moreover, the modelling approach is able to highlight the relation between the variables of different origins and the degree of their interconnectedness.

In the fourth topic dealing with muscle biochemistry and proteomics techniques, two original and independent papers are published [14,15]. The study by Zhu et al. [14] assess the usefulness of RNAlater^®^, regarded as a potential preservation method for proteins, to preserve *post-mortem* bovine muscle proteins compared with dry ice in a proteomic study. The protein profiles of muscle preserved in RNAlater^®^ were found, whatever the sampling time, to be similar to those of dry ice. The results demonstrate that RNAlater^®^ can be a simple and efficient way to preserve bovine muscle proteins for meat proteomics, where snap-freezing may not be a viable option for sample stabilization. The second study by Listrat et al. [15] deals with a deeper understanding of the ontogenesis of intramuscular connective tissue composition that would allow the control of muscle differentiation to improve beef quality. Therefore, the authors investigate the chronology of expression of ten extracellular matrix molecules in bovine *Semitendinosus* muscle using an immunohistology technique at five key stages of myogenesis. The data suggest that for the best controlling of the muscular differentiation to improve beef sensory quality, it would be necessary to intervene very early, i.e., at the beginning of the first-third of gestation.

In the fifth and last topic identified in this Special Issue, two original papers are categorised in the frame of consumer acceptability, development and characterisation of meat products. Čandek-Potokar et al. [16] aim to test the sensory acceptability of Slovenian consumers of a traditional product, the protected geographical indication dry-cured belly Kraška pancetta, produced from entire males, immunocastrates or surgical castrates. This preliminary study provides an overview of the sensory acceptability of dry-cured belly by Slovenian consumers in relation to gender, as characterized by leanness and boar taint level. The latest study by Burri et al. [17] tackles the subject of lipid oxidation known to affect the development and final qualities of meat and meat products [18]. Accordingly, the study by Burri et al. [17] had two main objectives: (i) develop a relevant oxidized processed meatball model to study the effects of supplemented antioxidants, and (ii) investigate lipid oxidation in meatballs without and with a range of eleven plant materials and extracts at different concentrations. The authors conclude that antioxidant-rich plant materials and extracts can efficiently prevent lipid oxidation in processed-meat products, such as meatballs.

In summary, the fifteen papers published in this Special Issue highlight a great part of the research activities in the field of meat science, aiming to characterize and improve the nutritional, sensory and physical quality of meat and meat products. This Special Issue feat, with the worldwide trend toward foods for nutrition and health, further states the importance of multidimensional and multidisciplinary approaches as exemplified in the papers described above. Finally, most authors who have contributed to this issue state that further research in their topic is required in every one of the presented papers and this assures an exciting time for future studies to improve and control the nutritional, sensory and physical quality aspects of meat and meat products.
